# Neopterin, Inflammation, and Oxidative Stress: What Could We Be Missing?

**DOI:** 10.3390/antiox7070080

**Published:** 2018-06-26

**Authors:** Steven P. Gieseg, Gregory Baxter-Parker, Angus Lindsay

**Affiliations:** 1 Free Radical Biochemistry Laboratory, School of Biological Sciences, University of Canterbury, Private Bag 4800, Christchurch 8140, New Zealand; greg.parker@pg.canterbury.ac.nz; 2 Department of Radiology, University of Otago Christchurch, Christchurch 8011, New Zealand; 3 Division of Rehabilitation Science and Division of Physical Therapy, Department of Rehabilitation Medicine, University of Minnesota, Minneapolis, MN 55455, USA; alindsay@umn.edu

**Keywords:** neopterin, inflammation, 7,8-dihydroneopterin, reactive-oxygen species, antioxidant

## Abstract

Neopterin has been extensively used as a clinical marker of immune activation during inflammation in a wide range of conditions and stresses. However, the analysis of neopterin alone neglects the cellular reactions that generate it in response to interferon-γ. Neopterin is the oxidation product of 7,8-dihydroneopterin, which is a potent antioxidant generated by interferon-γ-activated macrophages. 7,8-Dihydroneopterin can protect macrophage cells from a range of oxidants through a scavenging reaction that generates either neopterin or dihydroxanthopterin, depending on the oxidant. Therefore, plasma and urinary neopterin levels are dependent on both macrophage activation to generate 7,8-dihydroneopterin and subsequent oxidation to neopterin. This relationship is clearly shown in studies of exercise and impact-induced injury during intense contact sport. Here, we argue that neopterin and total neopterin, which is the combined value of 7,8-dihydroneopterin and neopterin, could provide a more comprehensive analysis of clinical inflammation than neopterin alone.

## 1. Introduction to Neopterin and 7,8-Dihydroneopterin

The level of clinical inflammation occurring as a result of physical trauma, cardiovascular disease, cancer, bacterial, parasitic infections, and viral infections is often assessed by measuring the concentration of plasma and urinary neopterin [1,2,3,4,5]. Neopterin analysis is used as the primary screen of blood donations in Australia as an indicator for safe blood transfusion [6]. Our own research group has made extensive use of neopterin analysis to assess exercise-induced injury and oxidative stress during in vitro cell culture [7,8,9]. The reason for neopterin’s popularity is threefold: neopterin is generated in response to γ-interferon activation of monocytes and macrophages, thus, it is a direct product of immune system activation [10,11]; neopterin is highly fluorescent, and thus easily detected at low concentration by HPLC [12,13]; there is a readily available ELISA-based assay for clinical use [14]. The rapid clearance of neopterinby the kidneys [15] also makes urinary neopterin a reliable measure of a person’s inflammatory state [9,16,17].

The convenience of measuring neopterin ignores the basic biology that macrophages do not enzymatically synthesize neopterin. Neopterin is an oxidized form of 7,8-dihydroneopterin, a product of γ-interferon-mediated upregulation of GTP cyclohydrolase I (GTPCH1) [18]. Therefore, the primary pterin generated in monocyte-derived macrophages during immune activation is 7,8-dihydroneopterin, not neopterin. An elevation in tissue and fluid neopterin concentrations are the result of both immune-activated macrophages, and oxidants reacting with 7,8-dihydroneopterin to generate neopterin. The consequence is that the ratio of neopterin to 7,8-dihydroneopterin may differ depending on the level of immune activation and oxidative environment at sites of inflammation. What we suggest should be measured clinically is neopterin and total neopterin (the combination of both neopterin and 7,8-dihydroneopterin) to gain a more accurate measurement of macrophage activity during inflammation [16]. The concept of measuring neopterin and total neopterin has become forgotten with the relative ease and convenience of measuring only neopterin. In this short review, we will examine the cellular biochemistry of 7,8-dihydroneopterin and neopterin to examine the potential additional benefit of measuring total neopterin as well as neopterin.

## 2. Synthesis within Macrophages

**Figure 1:**
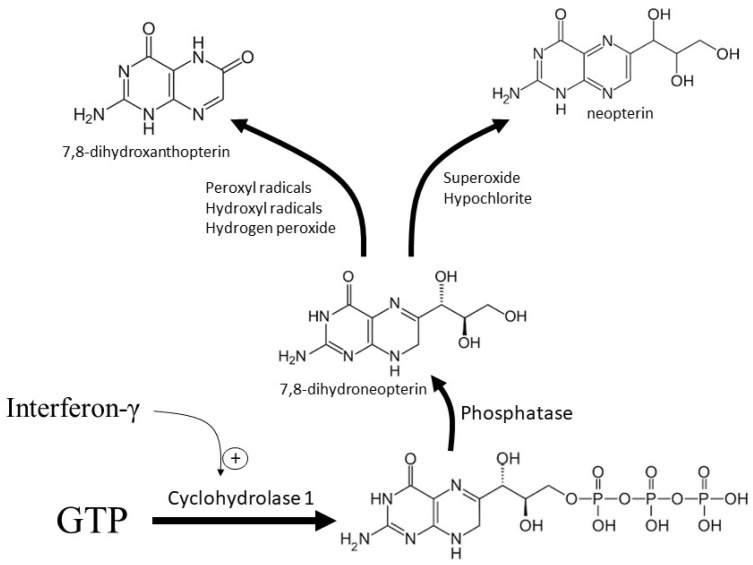
Formation and oxidation of 7,8-dihydroneopterin. In macrophages, interferon-γ upregulates the cytosolic enzyme GTP cyclohydrolase-1, which converts GTP to 7,8-dihydroneopterin-triphosphate. The action of non-specific phosphates generates free 7,8-dihydroneopterin whose oxidation generates neopterin or 7,8-dihydroxanthopterin depending on the oxidant.

Interferons α and γ, lipopolysaccharide, and possibly phorbol ester all upregulate the activity of monocyte-derived macrophage GTPCH1 [19]. GTPCH1 metabolizes GTP to 7,8-dihydroneopterin-triphosphate, which is then converted to 7,8-dihydroneopterin after dephosphorylation by non-specific phosphatases (Figure 1) [20]. In non-primate macrophage cells and other non-monocyte derived cells within the body, the combined enzymatic actions of 6-pyruvoyltetrahydropterin synthase followed by sepiapterin reductase converts 7,8-dihydroneopterin-triphosphate through to tetrahydrobiopterin, which is a key cofactor for a number of synthetic enzymes [21]. 7,8-Dihydroneopterin synthesis predominately occurs in primate macrophages because the expression of 6-pyruvoyltetrahydropterin synthase is not increased by interferon-γ. Thus, the main product of GTP metabolism by the GTPCH1 pathway becomes cytosolic 7,8-dihydroneopterin during macrophage activation in humans [18,21].

In turn, 7,8-dihydroneopterin and neopterin are readily transported in either direction across the cell membrane. In cell culture, the addition of 7,8-dihydroneopterin to an incubation media results in the relatively rapid accumulation of 7,8-dihydroneopterin within the cells [22].

## 3. Biological Activity of 7,8-Dihydroneopterin

7,8-Dihydroneopterin is a potent radical scavenging and chain-breaking antioxidant, which can out compete α-tocopherol for the lipid peroxyl radical during low-density lipoprotein (LDL) oxidation, even though it is water soluble [23]. Peroxyl and hydroxyl radical formation of lipid and protein hydroperoxides is inhibited by 7,8-dihydroneopterin, as well as the loss of protein thiols and glutathione [23,24,25]. Oxidant and cellular-mediated low-density lipoprotein oxidation is effectively inhibited by 7,8-dihydroneopterin [26,27]. Both the peroxyl radical and oxidized low-density lipoprotein (oxLDL) induced cell death in monocytes such as U937 cells, and human monocyte-derived macrophages are blocked by reducing the intracellular stress and preserving the cellular thiol levels in the presence of 7,8-dihydroneopterin [28,29,30]. The protective effects are due to the ability of 7,8-dihydroneopterin to rapidly scavenge the free radicals generated within the cellular environment, which is supported by reports indicating 7,8-dihydroneopterin scavenging hydroxyl and peroxyl radicals, HOCl, and possibly superoxide [25,29,31,32,33]. The product of these reactions is a neutralized oxidant or radical, due to the donation of electrons from 7,8-dihydroneopterin, and a range of 7,8-dihydroneopterin oxidation products, including neopterin (Figure 1). The reaction with hydroxyl and peroxyl radicals forms a minimal amount of neopterin, while the major product appears to be 7,8-dihydroxanthopterin [24,34,35]. Neopterin is the predominant product of the HOCl reaction, although the yield is not 100%, because neopterin also reacts with HOCl to generate non-pterin products [31,32]. Superoxide scavenging by 7,8-dihydroneopterin appears to generate neopterin based on cellular studies where NADPH-oxidase (NOX) is actively generating superoxide [29]. Interestingly, neopterin has been shown to inhibit NOX, suggesting a potential feedback loop during inflammation [36]. The antioxidant activity of 7,8-dihydroneopterin has led to the proposal that it is generated during macrophage activation with the purpose of self-protection within the highly oxidizing environment of an inflammatory site [23,37,38].

7,8-Dihydroneopterin, but not neopterin or xanthopterin, downregulates the level of CD36, which is the primary scavenger receptor responsible for the uptake of oxidized LDL and foam cell formation in atherosclerosis [29,39]. OxLDL uptake by macrophages via CD36 is unregulated, leading to the formation of lipid load foam cells within the atherosclerotic plaque. The downregulation of CD36 decreases oxLDL uptake in macrophages [29], suggesting that 7,8-dihydroneopterin may regulate to some extent foam cell formation within the artery wall.

Neopterin has been reported to activate inducible nitric oxide synthase (iNOS) in rat smooth muscle cells [40], but suppress iNOS activation in ovarian carcinoma cells [41], although the concentrations used were relatively high. 7,8-Dihydroneopterin also appears to have some cytotoxic effects, which are likely due to it being a strong reducing agent [42,43,44,45,46]. High concentrations of 7,8-dihydroneopterin and neopterin promote apoptosis in a number of cells, although we have not observed this with monocytes such as human-derived U937 or THP-1 cells, or with human monocyte-derived macrophages. 

The evidence suggests that measuring either neopterin or 7,8-dihydroneopterin alone cannot provide a complete picture of oxidative stress or immune activation. Rather, the combination of both biomarkers elucidates a more robust mechanism of the inflammatory process, with an analysis of neopterin being a measure of the oxidative status within the cells, and 7,8-dihydroneopterin being a measure of interferon-mediated cellular activation. Moreover, measuring the ratio between 7,8-dihydroneopterin and neopterin may elucidate differences in overall oxidative stress versus immune activation in different disease models and physical trauma.

## 4. Measurement of Neopterin and Total Neopterin

Neopterin was first isolated from human urine in 1967 [47] and used as a diagnostic biomarker of infection and illness [48]. Since then, there has been a plethora of analytical techniques developed and validated for the quantification of neopterin concentrations in a verity of bodily fluids. Serum and plasma neopterin are commonly measured to assess immune activation by radioimmuno assay (RIA), enzyme linked immunosorbent assay (ELISA) [49], or reverse phase high-performance liquid chromatography (RP-HPLC) coupled with fluorescence detection [50,51]. Levels of neopterin in urine have largely been quantified using RP-HPLC, in part due to the inaccuracy of RIA when analyzing urine [52]. However, ELISA is also an accurate tool for urinary neopterin measurement [14]. Neopterin has been quantified in other mediums including but not limited to, cerebrospinal fluid [53], cell media [18], and pus [54].

Although ELISA offers high throughput analysis within hospitals, small-scale clinical and research laboratory analysis have used HPLC. Since neopterin is highly fluorescent, nanomolar concentrations can be reliably detected after separation from other compounds. C18 has been popular, but we have found isocratic analysis on strong cation exchange (SCX) columns at pH 2.5 that were excellent for urine analysis [9,35]. However, for plasma analysis, we have found the amino column-based separation similarly reliable and efficient [55]. The amino column method was original designed for LC-MS, but we have found fluorescence detection to be more than adequate for most clinical needs.

Although an abundance of detection methodologies is promising, sample preparation still remains an important aspect. Since urine contains very low levels of proteins, and HPLC columns have become relatively inexpensive compared to sample clean-up costs, an HPLC analysis of urine usually involves the direct injection of diluted urine into the HPLC [9]. In comparison, plasma and cell lysates require complete protein removal before HPLC analysis. Solid phase extraction has often been used, but this adds considerable cost to the analysis [56]. Trichloroacetic acid precipitation has often be cited [12], but we found that significant neopterin is lost with this method when compared to protein precipitation with 50% acetonitrile [13]. 

In spite of 7,8-dihydroneopterin’s biosynthetic heritage providing potential for an excellent marker of immune activation, it has been widely neglected in clinical or experimental research. 7,8-Dihydroneopterin’s low fluorescence and nM concentration in plasma make it relatively difficult to detect. 7,8-Dihydroneopterin can be easily detected at μM concentration by its absorbance at 254 nm, but in clinical samples, it is usually at the nM level. There is also no ELISA kit that is commercially available for 7,8-dihydroneopterin, kits are only available for neopterin. 7,8-Dihydroneopterin is also both heat and UV light-sensitive in comparison to neopterin [57].

The usual approach to measure 7,8-dihydroneopterin is oxidation to neopterin using an acidic iodine solution [9,13,58] or manganese dioxide [17]. Therefore, the measurement of neopterin and 7,8-dihydroneopterin requires two injections of the sample: one untreated to measure neopterin, and the other where 7,8-dihydroneopterin has been oxidized to neopterin. The neopterin detected in an oxidized sample is a measure of the “total neopterin”, which is the combination of neopterin plus acidic iodide-oxidized 7,8-dihydroneopterin [9,16]. 7,8-Dihydroneopterin values can be calculated by subtracting neopterin from total neopterin. A key part of the oxidation methodology is regularly checking the acidic iodine solution to ensure complete oxidation of 7,8-dihydroneopterin to neopterin, because the solution does degrade with time.

Another reason that neopterin has been favored over total neopterin analysis is because 7,8-dihydroneopterin can be labile. In air-saturated solution at 25 °C, 7% of the 7,8-dihydroneopterin was lost over 4 h [57]. This rate of loss can be accelerated by UV light. If total neopterin is to be measured, it is important that samples are collected on ice and protected from UV light as much as possible. Care is also required to ensure that if samples are transported from a collection point to a site of analysis, there are tested procedures in place to eliminate the possibility of 7,8-dihydroneopterin oxidation. Overall, total neopterin analysis is more demanding to carry out than straight neopterin.

The two-step process to measure 7,8-dihydroneopterin can be avoided using HPLC coupled with mass spectrometry (LC-MS or LC-tandem mass spectrometry (LC-MS/MS)) [55,59]. The sensitivity of mass spectrometry has urinary neopterin and biopterin detection limits of 0.082 and 0.76 nM, respectively, which are much lower than fluorescence detection. Moreover, 7,8-dihydroneopterin has been directly detected and quantified by HPLC-MS, ameliorating the need for the oxidation step and second injection [59,60].

## 5. Clinical Effectiveness of Neopterin and Total Neopterin

While neopterin has been measured repeatedly as an assessment of macrophage activation and infiltration for several decades [5,48,61,62,63], complete estimation of immune activation through total neopterin analysis has limited transparency for clinical outcomes. In 1989, Fuchs et al. [16] measured neopterin plus 7,8-dihydroneopterin in HIV patients, suggesting that both provide equal potential for clinical diagnosis. Since 1989, neopterin and total neopterin have been measured selectively in various illnesses, including chronic renal failure [64] and HIV [61,65], which ultimately limits the diagnostic potential of neopterin and 7,8-dihydroneopterin as biomarkers due to a lack of specificity for any particular inflammatory condition. A recent study has measured urinary neopterin and 7,8-dihydroneopterin in patients with the chronic inflammatory disease Duchenne muscular dystrophy (DMD) [66]. Lindsay et al. [67] measured variable differences in neopterin based on hydration correction methods, but a significant elevation in 7,8-dihydroneopterin in DMD patients compared with healthy age-matched controls. Thus, urinary neopterin analysis would have suggested that DMD patients do not have elevated immune activation, and solidifies the need for neopterin and total neopterin analysis in a clinical environment.

Recently, neopterin and 7,8-dihydroneopterin have been used as indicators of immune system activation in sport and exercise medicine, and are gaining momentum over other traditional inflammatory markers [68]. Their benefits include non-invasive urinary assessment, economical analysis by HPLC [9], and fast elimination kinetics [7] that offer immediate and cost-effective analysis; these advantages are pertinent in exercise stress evaluation and athlete management. In a clinical setting, total neopterin is still under-represented when examining diseases and illnesses of an inflammatory nature. However, evidence suggests that participants subjected to high intensity and trauma-inducing exercise stimulate 7,8-dihydroneopterin production, but with varying degrees of its oxidized form.

Neopterin has been routinely measured in several high-intensity exercise studies. Neopterin has been shown to rise in response to high-intensity cycling [69], ultra-endurance events [70], body-building [9], mixed martial arts [8,71,72], and professional rugby [35,73,74], and has shown promise as an indicator of non-functional overreaching or over-training syndrome [75]. Contrasting results suggest that its accumulation may be intensity-dependent [76,77], as the impacts and total running distance during a game of rugby strongly correlate with the observed increase in neopterin and total neopterin concentration [73]. Recently, neopterin has also been used to assess the positional demands of professional rugby players [78], and used to evaluate the effectiveness of post-game rugby union recovery interventions [79], the efficacy of cold water immersion [1,2,3], stress perturbations associated with ischemic preconditioning [4], and the monitoring of season-long stress fluctuations in professional rugby players [5].

Typically, exercise studies have only measured neopterin [6,7]. Whilst neopterin provides an estimation of immune system activation, research has widely neglected the measurement of 7,8-dihydroneopterin, which is critical for understanding total macrophage activation following exercise and trauma. The measurement of neopterin alone may simply provide an estimation of the change in oxidative status of an individual. However, research has observed significant increases in other inflammatory mediators such as C-reactive protein and TNF-α in conjunction with neopterin following muscle damaging exercise, albeit at various time points [8].

Recent studies examining impact-induced trauma during exercise have begun to elucidate the importance of measuring neopterin in addition to 7,8-dihydroneopterin. The combined analysis provides a comprehensive overview of exercise-induced changes in oxidative status and immune system activation [35,71,72]. For example, a study measuring changes in neopterin and 7,8-dihydroneopterin following a mixed martial arts training session has shown that 7,8-dihydroneopterin can dramatically increase without any change in neopterin [8]. There is also evidence that the ratio of neopterin to total neopterin can range from 20.9–92.1% [9]. Within atherosclerotic plaque, we have observed a section of plaque that contains only 7,8-dihydroneopterin, while other sections only contain neopterin [80]. Moreover, extreme intra individual and inter individual variation exists in athletes following high-intensity exercise [8,73], thus cementing the need for the measurement of both compounds to separate changes in oxidative status and immune system activation.

To complicate the exercise-related research on neopterin, recent evidence [35,71,72] has identified a correlation between impact-induced myoglobin release and changes in neopterin concentration. Hypochlorite, which is produced primarily by neutrophils and macrophages to a lesser extent [81,82], is capable of oxidizing 7,8-dihydroneopteirn to neopterin in vivo [31,32]. There is also evidence that superoxide can also oxidize 7,8-dihydroneopterin to neopterin, as the addition of apocynin to macrophages inhibits the oxidation of 7,8-dihydroneopterin to neopterin [29]. The in vitro and in vivo oxidation of 7,8-dihydroneopterin to neopterin by myoglobin verifies its previously identified oxidative potential [83,84]. This suggests that muscle-damaging exercise resulting in the release of intracellular constituents may exacerbate oxidative stress (neopterin), resulting in significant elevations that may not be directly related to the acute phase response. This further substantiates the necessity of neopterin and 7,8-dihydroneopterin measurement for separating and accurately disseminating oxidative stress and inflammation-related exercise research.

## 6. Conclusions

Collectively, sport and exercise research that have measured neopterin and 7,8-dihydroneopterin have provided evidence that the change in one does not necessarily result in a change in the other. To critically and correctly evaluate a clinical outcome, treatment efficacy, or the oxidative status/immune system activation of an individual, neopterin and 7,8-dihydroneopterin should be simultaneously measured. There is a clear need to bring the analysis of neopterin and 7,8-dihydroneopterin from sport and exercise medicine back into the classical clinical environment. The measurement of neopterin and total neopterin would provide a clearer and more sensitive measure of patient oxidative stress and inflammation during clinical events.
